# A novel strategy of “pick the best of the best” for the nondestructive identification of Poria cocos based on near‐infrared spectroscopy

**DOI:** 10.1002/fsn3.2383

**Published:** 2021-06-19

**Authors:** Jiayi Li, Mei Yu, Shangke Li, Liwen Jiang, Yu Zheng, Pao Li

**Affiliations:** ^1^ College of Food Science and Technology Hunan Provincial Key Laboratory of Food Science and Biotechnology Hunan Agricultural University Changsha China; ^2^ Hunan Agricultural Product Processing Institute Hunan Academy of Agricultural Sciences Changsha China; ^3^ School of Medicine Hunan Normal University Changsha China

**Keywords:** near‐infrared spectroscopy, nondestructive analysis, pick the best of the best, poria cocos, principal component analysis

## Abstract

In this paper, a novel strategy of “pick the best of the best” was proposed for the nondestructive identification of different‐origin and adulterated Poria cocos with near‐infrared spectroscopy. First, various preprocessing methods were divided into three classes: baseline correction, scattering and trend correction, and scaling. The single preprocessing methods with the best predictions in each class were selected. Then, the selected preprocessing methods were combined in pairs according to three classes. The pair combination preprocessing methods with the best predictions and also better predictions than single methods were selected. Finally, the selected pair combination preprocessing method was combined with the methods in the unselected class. The three combination preprocessing methods with the best predictions and also better predictions than pair combination methods were selected as the final prediction. With this strategy, the optimized preprocessing combination can be obtained quickly, and the identification accuracy with principal component analysis method can be greatly improved. 0% identification accuracy of adulterated samples and 12.5% identification accuracy of different‐origin samples were obtained with the raw data. However, 100% accuracy of adulterated samples, 93.8% accuracy of calibration dataset, and 75% accuracy of validation dataset can be obtained with the novel strategy. The developed technology can be regarded as a simple, rapid, and accurate nondestructive identification method for different‐origin and adulterated samples, and has a broad application prospect in the future.

## Graphical abstract

A novel strategy of ‘pick the best of the best’ was proposed for the nondestructive identification of different‐origin and adulterated Poria cocos with near infrared spectroscopy. 100% accuracy of adulterated samples, 93.8% accuracy of calibration dataset and 75% accuracy of validation dataset can be obtained with the novel strategy.

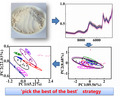

## 1 INTRODUCTION

Poria cocos is the dried sclerotia of Poria cocos (Schw.) Wolf, and has been used for several thousand years as a kind of food and medicine function (Tian et al., 2019). The most commonly consumed product of Poria cocos is Poria cocos powder. The main active ingredients of Poria cocos include pachyman, poriatin, triterpenoids, etc. It is also rich in amino acids, choline, and potassium salt. Modern pharmacological studies have shown that Poria cocos has the effects of liver protection, immune regulation, anti‐tumor, anti‐oxidation, anti‐inflammatory, and anti‐virus (Khan et al., 2018; Lee et al., 2018; Miao et al., 2016). The active substances of Poria cocos are easily affected by planting region. The research shows that the chemical components of Poria cocos from different producing areas are not same, while the efficacy and drug properties are also different (Y. Li et al., 2016; Wang et al., 2013). However, the difference of Poria cocos from different producing areas is not significant in appearance and is difficult to be identified by laymen. In recent years, some illegal businessmen used starch as Poria cocos powder or mixed starch into Poria cocos powder to seek illegal interests (Chang et al., 2019). However, it is not easy to distinguish Poria cocos powder from adulterated samples. Various analytical technologies have been used for the analysis of Poria cocos samples (K. Li et al., 2013; Y. Li et al., 2016; Zhu et al., 2018). For example, ultra‐high performance liquid chromatography‐ultraviolet‐tandem mass spectrometry (UPLC‐UV‐MS) was applied for the analysis of the fingerprint of triterpenoid constituents in Poria cocos (K. Li et al., 2013). A quality assessment system comprised of a tandem technique of ultraviolet spectroscopy and ultra‐fast liquid chromatography (UFLC) was applied for the analysis of Poria cocos in different regions of Yunnan (Y. Li et al., 2016). The result showed that the composition and content of polysaccharides and triterpenoids in Poria cocos from different areas are different. In addition, the content of polysaccharides in different medicinal parts is also different. However, most of these methods are destructive, time‐consuming, and laborious. Besides, the research on the identification of Poria cocos adulteration is basically in the blank. Therefore, it is urgent to develop a rapid and nondestructive method for the identification of Poria cocos from different producing areas and adulterated samples.

Near‐infrared spectroscopy (NIRS) technology has been widely used in the rapid and nondestructive analysis of agricultural (Tardaguila et al., 2017), food (P. Li et al., 2020; P. Li et al., 2019) and pharmaceutical samples (P. Li et al., 2012), which is based on overtones and combinations of fundamental vibrations from the H‐containing group. However, due to the complexity of the samples, the useful information is usually carried by broad spectral peaks. Besides, the spectra are often disturbed by stray light, noise, and baseline drift interferences. In order to eliminate the background and noise interference in the spectra, a large number of spectral preprocessing methods have been proposed before modeling, including de‐bias correction, detrend (DT), maximum and minimum normalization (MinMax), standard normal variate (SNV) transformation, multiplicative scatter correction (MSC), first‐order derivative (1st Der), second‐order derivative (2nd Der), and continuous wavelet transform (CWT) (Bian et al., 2016; Han et al., 2017; Ma et al., 2020). De‐bias correction and DT algorithms are used to eliminate baseline drift. MinMax method is the most frequently used scaling technique that normalizes all variables to a certain range. SNV and MSC methods can be used to eliminate the light scattering effect due to uneven distribution of particle and different particle size. CWT and derivative methods can subtract the influence of background or drift in the signal. The resolution and sensitivity of the spectra can also be improved. However, the signal noise ratio may decrease in the same time. In order to eliminate multiple interferences in the spectra, a combination of various preprocessing methods is often needed (Bian et al., 2020). How to choose the best preprocessing method and its combination is a problem that must be considered. Visual inspection (Engel et al., 2013) and trial‐and‐error strategy (Chen & Grant, 2012) are the most used preprocessing selection method. However, the strategies are time‐consuming especially for the analysis of large dataset.

The aim of this study is to develop an effective way to find the optimized preprocessing method and establish a nondestructive method for the identification of different‐origin and adulterated Poria cocos. Besides, to the best of our knowledge, there is no report on simultaneous identification of different‐origin and adulterated Poria cocos. Spectra of Poria cocos powder samples were obtained directly by the NIRS instrument. The optimized preprocessing method was found by a novel strategy of “pick the best of the best” quickly. Principal component analysis (PCA) combined with the optimized preprocessing methods were used for the identification of different‐origin and adulterated Poria cocos.

## 2 MATERIALS AND METHODS

### 2.1 Poria cocos sample

**FIGURE 1:**
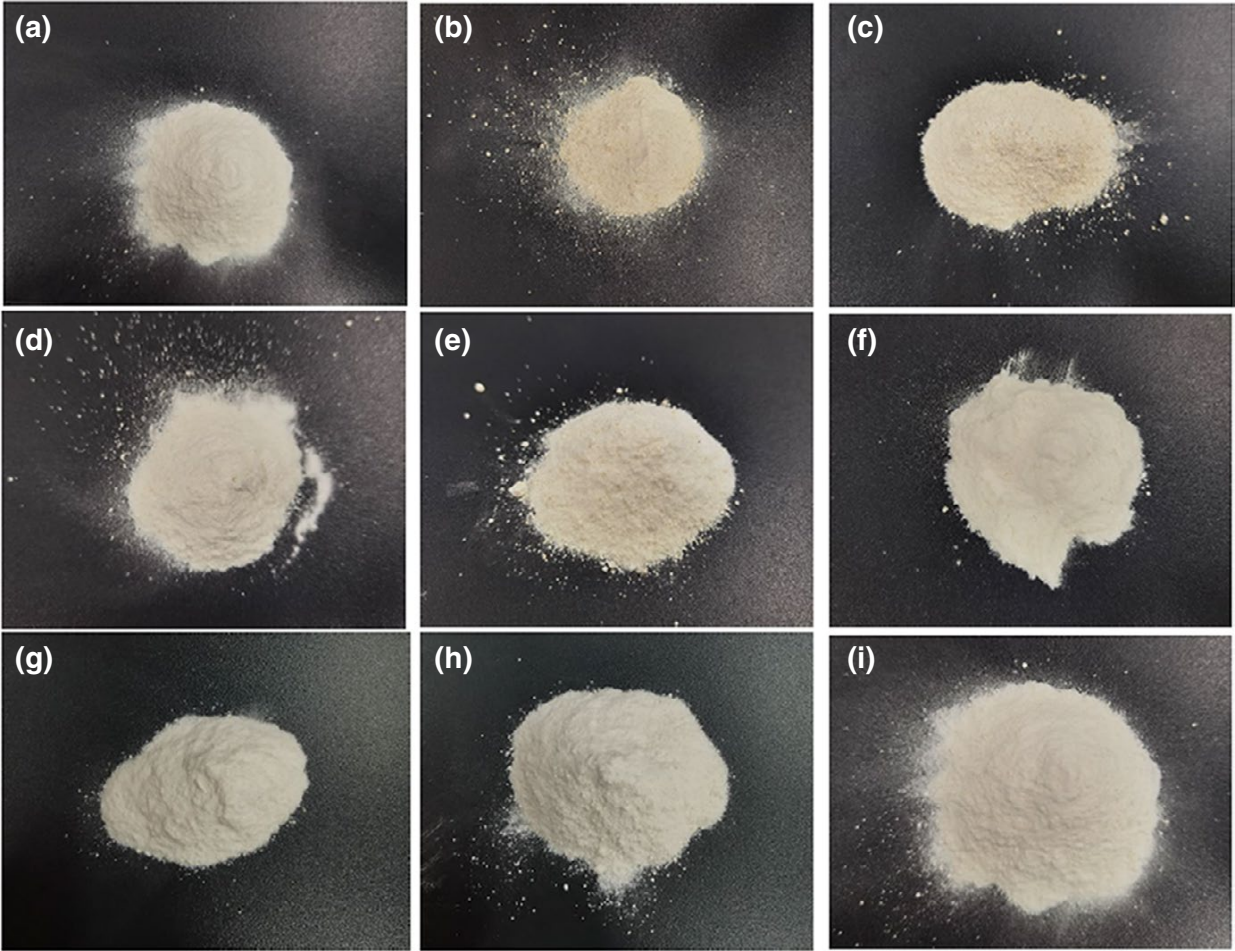
Pictures of the samples. (a)‐(i): samples in Anhui, Fujian, Guangxi, Guizhou, Henan, Hunan, Sichuan, Yunnan, and adulterated samples

Poria cocos powder samples from different areas were purchased from several local pharmacy shops, which were produced in Anhui, Fujian, Guangxi, Guizhou, Henan, Hunan, Sichuan, and Yunnan. All samples have similar appearances and the colors. Ten samples were taken from each group and a total of 80 samples were collected. Poria cocos may be adulterated by starch adulterants due to the similar color. Therefore, Poria cocos powder samples from Yunnan were adulterated by starch. The ratios of Poria cocos and starch were 1:0.25, 1:0.5, 1:0.75, and 1:1. Four samples were taken from each group and a total of 16 adulterated samples were collected. The pictures of the samples were shown in Figure 1. The differences of Poria cocos from different producing areas and adulterated samples are not significant in appearance, and are difficult to be identified by laymen.

### 2.2 Instrumentation and measurements

The spectra were obtained directly by the Antaris II NIRS instrument (ThermoFisher, USA) in diffuse reflectance mode with integrating sphere diffuse reflection accessory. The powder samples were placed in a quartz sample bottle and measured by directly placing the sample bottle at the center of the light spot. To increase signal‐to‐noise ratio (SNR), all reference and sample spectra were measured with scan number 128. The measurements were repeated three times and averaged. Each spectrum is composed of 1557 data points recorded from 10,000 to 4,000 cm^−1^. The resolution of the instrument is 4 cm^‐1^.

### 2.3 Data analysis

In order to eliminate the background and noise interference in the spectra, spectral preprocessing methods, including DT, de‐bias, SNV transformation, MinMax, MSC, 1st Der, 2nd Der, and CWT methods, were used for spectra pretreatment before PCA calculation. In the calculations of CWT method, “haar” wavelet and scale =20 were adopted. In order to eliminate multiple interferences in the spectra, combination of various preprocessing methods was used. How to choose the best preprocessing method and its combination is a problem that must be considered. However, it is time‐consuming to get the best pretreatment combination by random combination. A total of 109,601 preprocessing methods and their combinations (9 + 8×7 + 8×7 × 6+8 × 7×6 × 5^…^+8 × 7×6 × 5×4 × 3×2 × 1) can be obtained, including no preprocessing, one to eight preprocessing combinations. The strategies are time‐consuming especially for the analysis of large dataset. In this paper, the best preprocessing method was selected by employing the principle “pick the best of the best.” First, according to the literature (Bian et al., 2020; Gerretzen et al., ,^2015^, ^2016^), eight preprocessing methods were divided into three classes: baseline correction, scattering and trend correction, and scaling, shown in Table 1. CWT and derivative methods (1st Der and 2nd Der) are baseline correction methods which can subtract the influence of background or drift in the signal. SNV and MSC methods are two well‐known scatter correction methods which can eliminate the light scattering effect due to uneven distribution of particle and different particle size. MinMax method is the most frequently used scaling technique that normalizes all variables to a certain range. The preprocessing methods with the best predictions in each class were selected. Then, the selected preprocessing methods were combined in pairs according to the three classes. The pair combination preprocessing methods with the best predictions and also better predictions than single methods were selected. Finally, the selected pair combination preprocessing methods were combined with the methods in the unselected class. The three combination preprocessing methods with the best predictions and also better predictions than pair combination method were selected as the final prediction. A total of 119 preprocessing methods and their combinations can be obtained, shown in Table 2.

**TABLE 1:** Three types of preprocessing methods

Baseline correction	Scatter and trend correction	Scaling
1st Der	De‐bias	MinMax
2nd Der	DT	
CWT	SNV	
	MSC	

**TABLE 2:** 119 preprocessing methods

No.	Methods	No.	Methods	No.	Methods	No.	Methods
1	none	31	DT+CWT	61	1st Der+MinMax+DT	91	SNV+MinMax+2nd Der
2	1st Der	32	DT+MinMax	62	1st Der+MinMax+SNV	92	SNV+MinMax+CWT
3	2nd Der	33	SNV+1st Der	63	1st Der+MinMax+MSC	93	MSC+MinMax+1st Der
4	CWT	34	SNV+2nd Der	64	2nd Der+MinMax+De‐bias	94	MSC+MinMax+2nd Der
5	De‐bias	35	SNV+CWT	65	2nd Der+MinMax+DT	95	MSC+MinMax+CWT
6	DT	36	SNV+MinMax	66	2nd Der+MinMax+SNV	96	MinMax+1st Der+De‐bias
7	SNV	37	MSC+1st Der	67	2nd Der+MinMax+MSC	97	MinMax+1st Der+DT
8	MSC	38	MSC+2nd Der	68	CWT+MinMax+De‐bias	98	MinMax+1st Der+SNV
9	MinMax	39	MSC+CWT	69	CWT+MinMax+DT	99	MinMax+1st Der+MSC
10	1st Der+De‐bias	40	MSC+MinMax	70	CWT+MinMax+SNV	100	MinMax+2nd Der+De‐bias
11	1st Der+DT	41	MinMax+1st Der	71	CWT+MinMax+MSC	101	MinMax+2nd Der+DT
12	1st Der+SNV	42	MinMax+2nd Der	72	De‐bias+1st Der+MinMax	102	MinMax+2nd Der+SNV
13	1st Der+MSC	43	MinMax+CWT	73	De‐bias+2nd Der+MinMax	103	MinMax+2nd Der+MSC
14	1st Der+MinMax	44	MinMax+De‐bias	74	De‐bias+CWT+MinMax	104	MinMax+CWT+De‐bias
15	2nd Der+De‐bias	45	MinMax+DT	75	DT+1st Der+MinMax	105	MinMax+CWT+DT
16	2nd Der+DT	46	MinMax+SNV	76	DT+2nd Der+MinMax	106	MinMax+CWT+SNV
17	2nd Der+SNV	47	MinMax+MSC	77	DT+CWT+MinMax	107	MinMax+CWT+MSC
18	2nd Der+MSC	48	1st Der+De‐bias+MinMax	78	SNV+1st Der+MinMax	108	MinMax+De‐bias+1st Der
19	2nd Der+MinMax	49	1st Der+DT+MinMax	79	SNV+2nd Der+MinMax	109	MinMax+De‐bias+2nd Der
20	CWT+De‐bias	50	1st Der+SNV+MinMax	80	SNV+CWT+MinMax	110	MinMax+De‐bias+CWT
21	CWT+DT	51	1st Der+MSC+MinMax	81	MSC+1st Der+MinMax	111	MinMax+DT+1st Der
22	CWT+SNV	52	2nd Der+De‐bias+MinMax	82	MSC+2nd Der+MinMax	112	MinMax+DT+2nd Der
23	CWT+MSC	53	2nd Der+DT+MinMax	83	MSC+CWT+MinMax	113	MinMax+DT+CWT
24	CWT+MinMax	54	2nd Der+SNV+MinMax	84	De‐bias+MinMax+1st Der	114	MinMax+SNV+1st Der
25	De‐bias+1st Der	55	2nd Der+MSC+MinMax	85	De‐bias+MinMax+2nd Der	115	MinMax+SNV+2nd Der
26	De‐bias+2nd Der	56	CWT+De‐bias+MinMax	86	De‐bias+MinMax+CWT	116	MinMax+SNV+CWT
27	De‐bias+CWT	57	CWT+DT+MinMax	87	DT+MinMax+1st Der	117	MinMax+MSC+1st Der
28	De‐bias+MinMax	58	CWT+SNV+MinMax	88	DT+MinMax+2nd Der	118	MinMax+MSC+2nd Der
29	DT+1st Der	59	CWT+MSC+MinMax	89	DT+MinMax+CWT	119	MinMax+MSC+CWT
30	DT+2nd Der	60	1st Der+MinMax+De‐bias	90	SNV+MinMax+1st Der		

Eighty samples were divided into a calibration dataset with 64 samples and a validation dataset with 16 samples by Kennard‐Stone (KS) method. In order to discriminate the Poria cocos powder samples from different producing areas and adulterated samples, PCA combined with single and combined pretreatment method was performed. When the sample data point is labeled in the confidence ellipse of this category and not in the confidence ellipses of other categories, the identification of the sample category is accurate. When the adulterated sample data point is not labeled in the confidence ellipse of any category, the identification of the adulterated sample is accurate.

The programs were performed using Matlab 2010a (The Mathworks, USA) and run on a personal computer.

## 3 RESULTS AND DISCUSSION

### 3.1 Identification of Poria cocos with single preprocessing techniques

**FIGURE 2:**
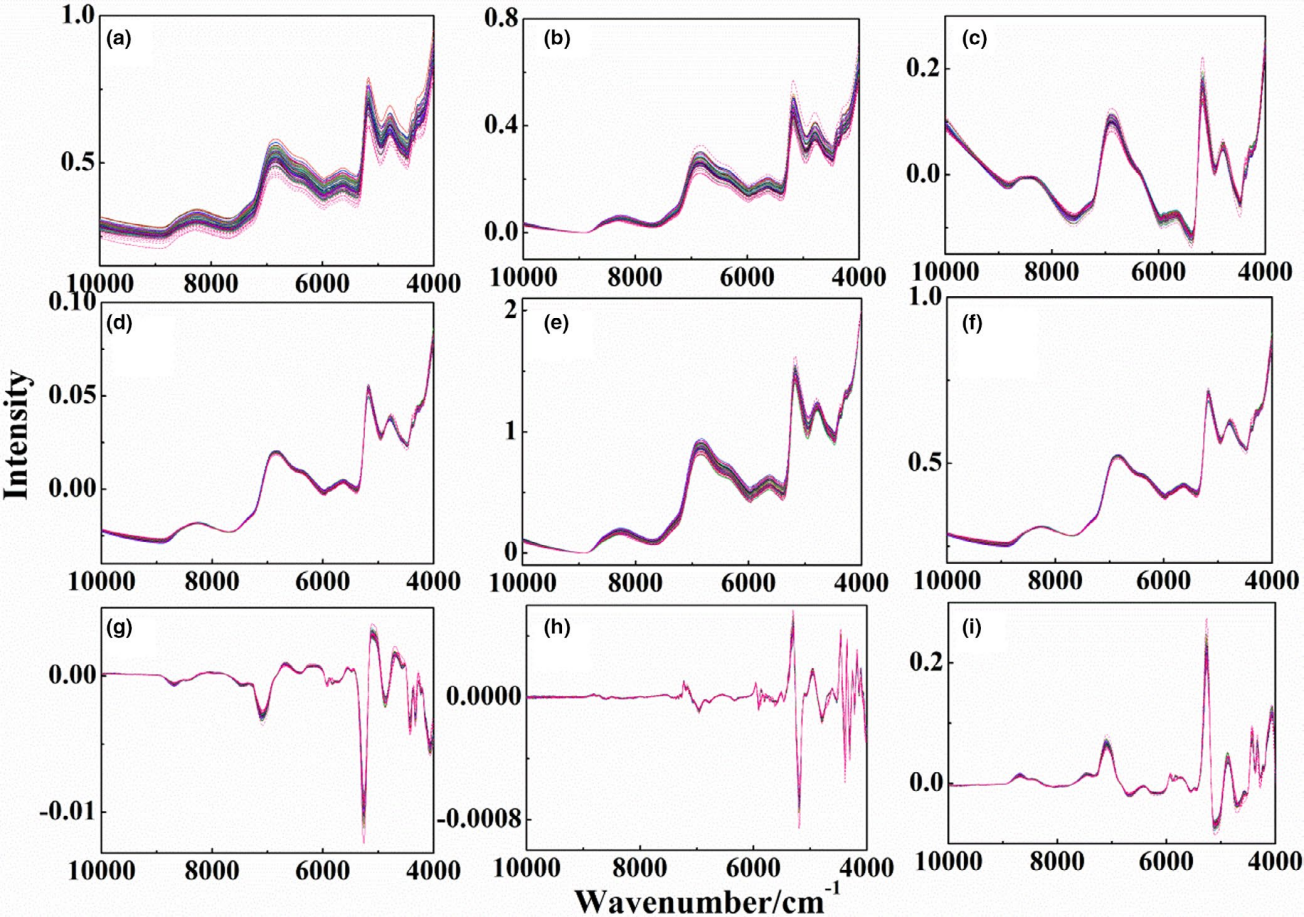
Spectra of Poria cocos powder and adulterated samples with single preprocessing methods, (a)‐(i): raw data, de‐bias, DT, SNV transformation, MinMax, MSC, 1st Der, 2nd Der, and CWT

Figure 2(a) shows the original spectra of different‐origin and adulterated Poria cocos. The adulterated samples were marked with red lines. There was very obvious baseline drift interference in the spectra. Besides, there were no apparent differences in the raw spectra due to severe overlapping. It is difficult to find the difference between the spectra of samples from different producing areas and adulterated samples. Each spectrum has six groups of peaks in the wavenumber range of 8800–7600, 7100–6000, 6000–5400, 5400–4900, 4900–4500, and 4450–4150 cm^−1^, which belong to CH second overtone bands, OH first overtone bands, CH first overtone bands, OH combination bands, NH and OH combination bands, and CH combination bands, respectively. The results are basically same with the former literature (Yi et al., 2020). Figure 2(b)‐(i) show the spectra with de‐bias, DT, SNV transformation, MinMax, MSC, 1st Der, 2nd Der, and CWT methods, respectively. Compared with the spectra of raw data, the baseline drift interference can be reduced with de‐bias, DT, and MinMax methods. The baseline drift interference can be further eliminated by SNV transformation and MSC methods. The influence of background and baseline drift can be effectively eliminated by CWT and derivative methods. However, it is still difficult to find the difference between the spectra of samples from different producing areas and adulterated samples.

**FIGURE 3:**
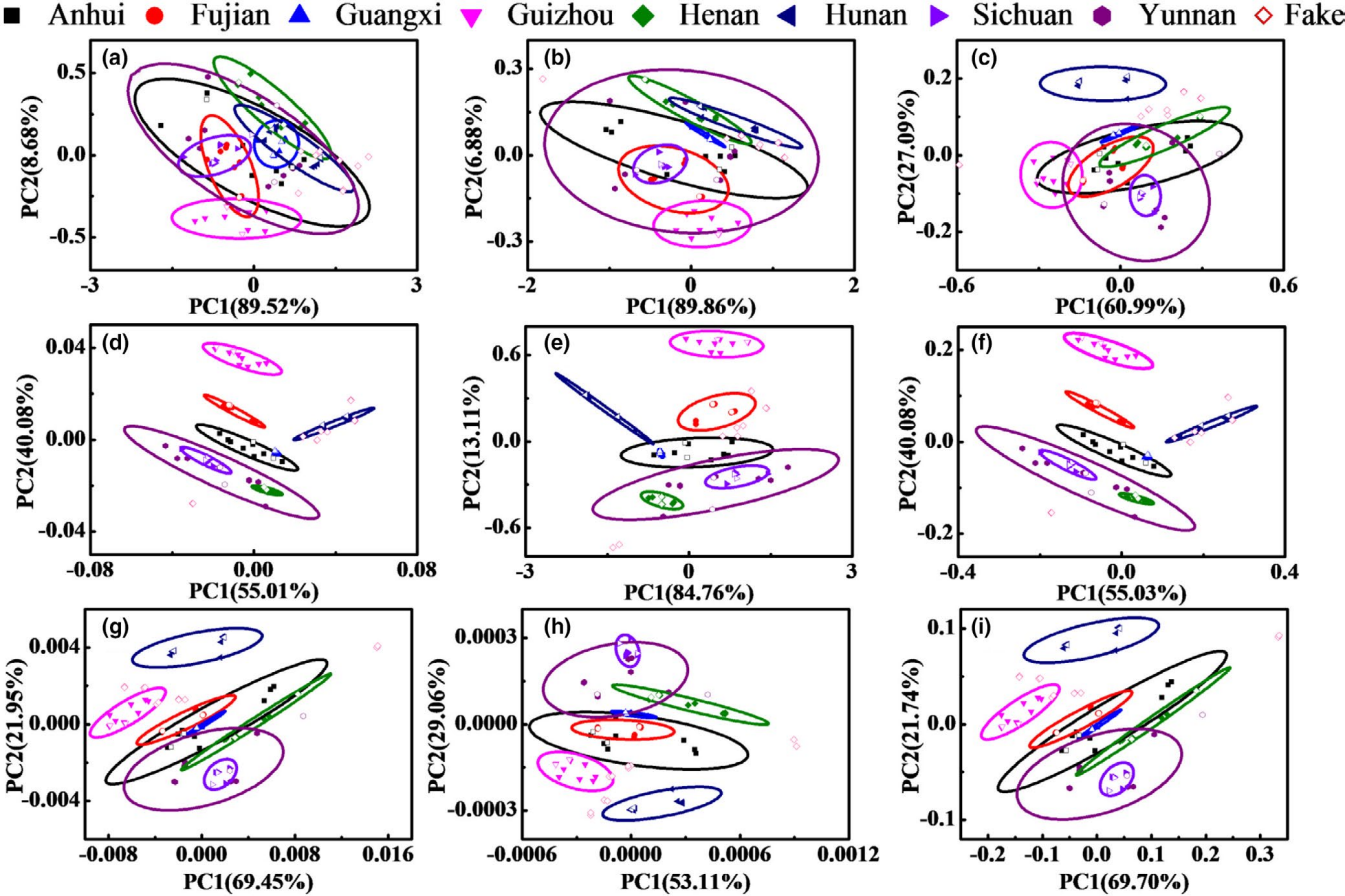
PCA results of Poria cocos powder and adulterated samples with single preprocessing methods, (a)‐(i): raw data, de‐bias, DT, SNV transformation, MinMax, MSC, 1st Der, 2nd Der, and CWT

**FIGURE 4:**
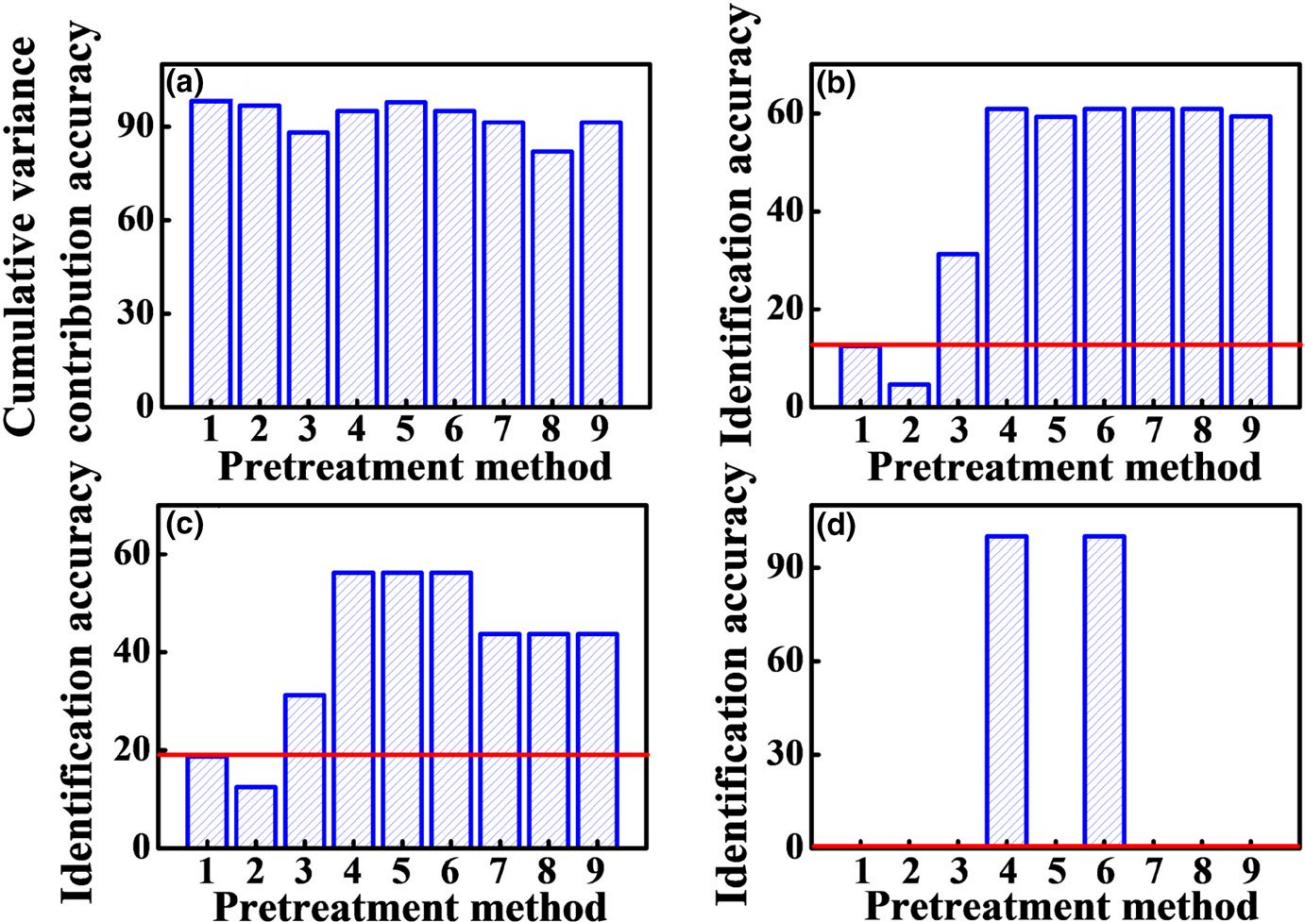
The cumulative variance contribution rate of first two scores (a), the identification accuracies of calibration dataset (b), validation dataset (c), and adulterated samples by single preprocessing methods. The red line is the accuracy with the raw data

In order to discriminate the Poria cocos powder samples from different producing areas and adulterated samples, PCA method was performed. Figure 3 shows the PCA results with different single preprocessing methods. The validation samples were marked with hollow icons and adulterated samples were marked with red icons. Figure 4 shows the cumulative variance contribution rate of the first two scores, the identification accuracies of calibration dataset, validation dataset, and adulterated samples by single preprocessing methods. The position of red line indicates the identification accuracy with no preprocessing. It can be clearly seen that the performance with single preprocessing methods was enhanced except de‐bias method, compared with the result of the raw data. The best accuracy of calibration dataset is 60.9% with the SNV transformation, MSC, 1st Der, and 2nd Der methods, while the best accuracy of validation dataset is 56.3% with the SNV, MSC, and MinMax methods. The best identification accuracy of adulterated samples is 100.0% with the SNV transformation and MSC methods. It is because that SNV transformation and MSC methods can eliminate the light scattering effect due to uneven distribution of particle and different particle size. The above results show that SNV transformation and MSC are the best, followed by MinMax, CWT and derivative methods, DT, and de‐bias methods. The preprocessing methods with the best predictions in each class were selected. Therefore, the preprocessing methods of SNV transformation, MSC, MinMax, 1st Der, and 2nd Der were selected for the next calculation.

### 3.2 Identification of Poria cocos by pair combination preprocessing methods with “pick the best of the best” strategy

**FIGURE 5:**
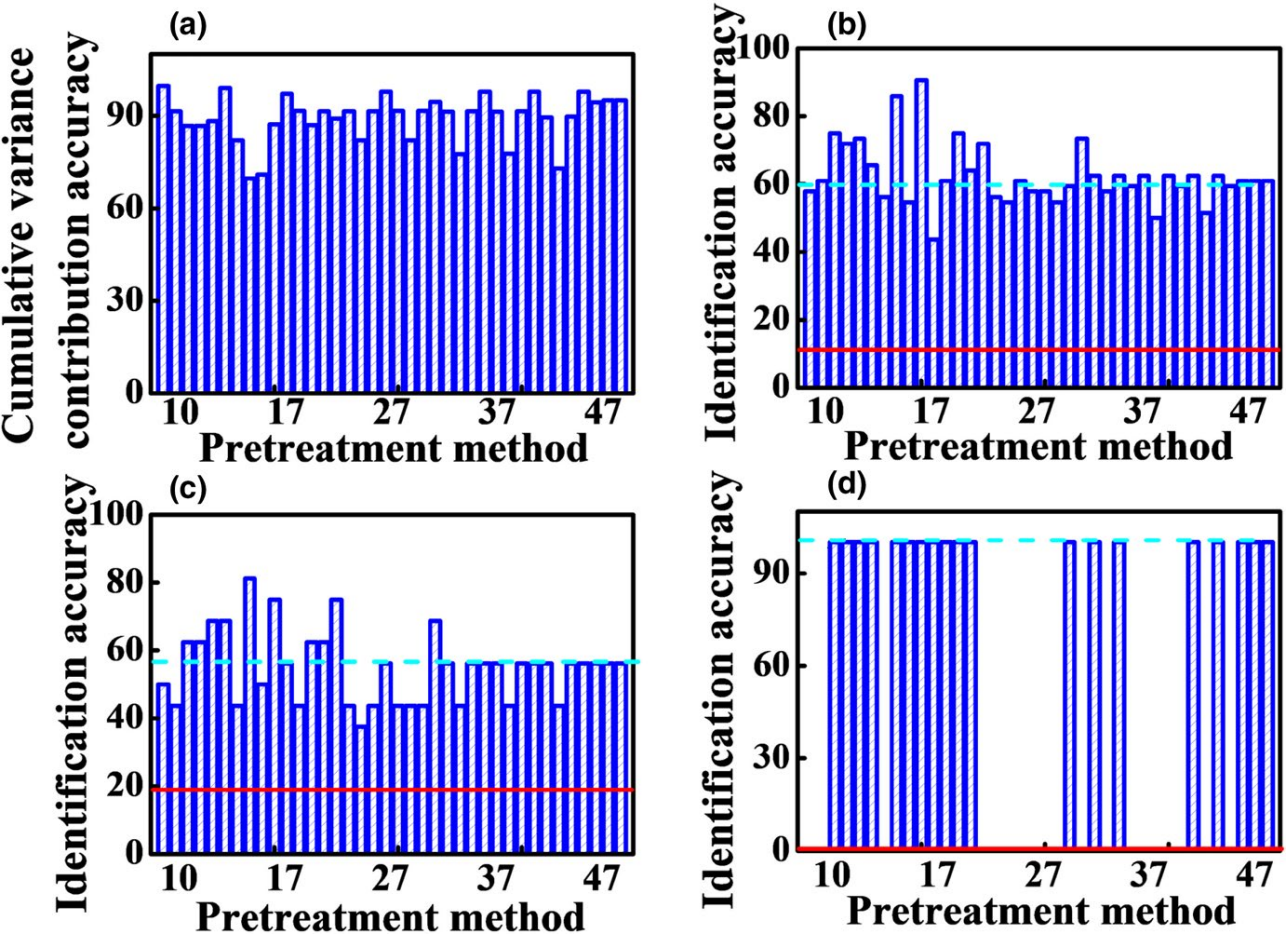
The cumulative variance contribution rate of first two scores (a), the identification accuracies of calibration dataset (b), validation dataset (c), and adulterated samples (d) by pair combination preprocessing methods. The red line is the accuracy with the raw data, and the blue dash line is the accuracy with the best single preprocessing methods

The best preprocessing method was selected by employing the principle “pick the best of the best.” The selected single preprocessing methods were combined in pairs according to the three classes. Therefore, methods of Nos. 12–14, 17–19, 33, 34, 36–38, 40–42, 46, and 47 were selected. Figure 5 shows the cumulative variance contribution rate of the first two scores, the identification accuracies of calibration dataset, validation dataset, and adulterated samples by pair combination preprocessing methods. The pair combination preprocessing strategy improves the identification performance. The best accuracy of calibration dataset is 90.6% with the 2nd Der+MinMax methods. Although the accuracy of validation dataset with 2nd Der+SNV is the highest (81.3%), the cumulative variance contribution rate of first two scores is less than 70% and the results are unreliable. The best accuracy of validation dataset is 75.0% with the 2nd Der+MinMax and CWT+MinMax methods. The 100.0% identification accuracy of the adulterated samples can also be obtained with the 2nd Der+MinMax method. Therefore, 2nd Der+MinMax method is the best pair combination preprocessing method. In addition, the results with different combination order are different. The accuracies with CWT/derivative+MinMax method are better than MinMax+CWT/derivative method, while the accuracies with CWT/derivative+SNV/MSC method are better than SNV/MSC+CWT/derivative method. MinMax+SNV/MSC method are better than SNV/MSC+MinMax method. Besides, the combination with unselected methods of DT or de‐bias cannot improve the identification accuracy. The result shows that the preprocessing strategy is feasible and effective. The pair combination preprocessing method with the best predictions and also better predictions than single method was selected. Therefore, the preprocessing method of 2nd Der+MinMax was selected for the next calculation.

### 3.3 Identification of Poria cocos by three combination preprocessing methods with “pick the best of the best” strategy

**FIGURE 6:**
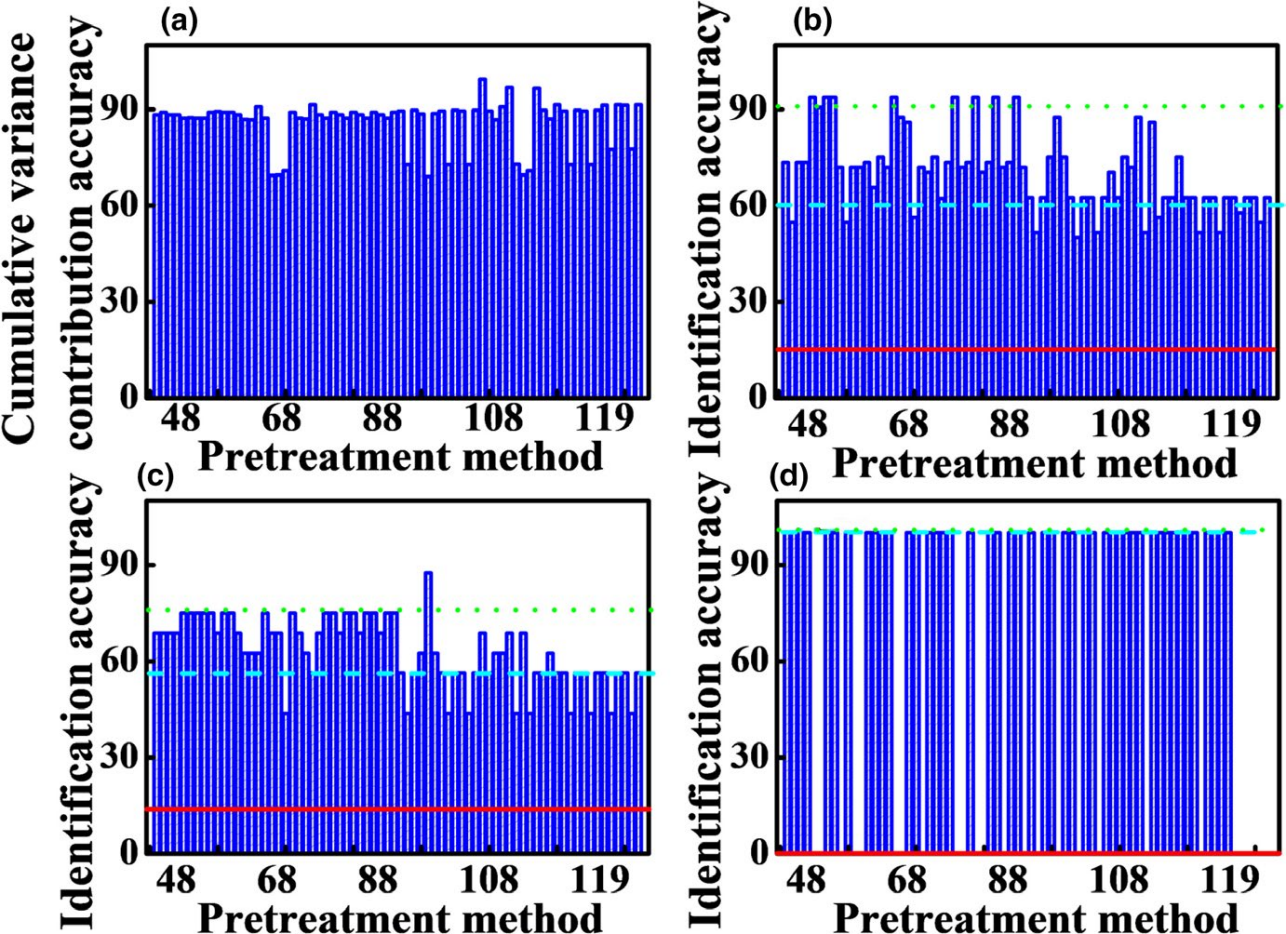
The cumulative variance contribution rate of first two scores (a), the identification accuracies of calibration dataset (b), validation dataset (c), and adulterated samples (d) by three combination preprocessing methods. The red line is the accuracy with the raw data. The blue dash line is the accuracy with the best single preprocessing methods. The green dot line is the accuracy with the best pair preprocessing methods

Combination of three preprocessing techniques with “pick the best of the best” strategy was applied to further improve the accuracy. Based on the section 3.2, the selected 2nd Der+MinMax method was combined with the methods in the unselected class. Therefore, methods of Nos. 66, 67, 54, 55, 79, and 82 were selected. Figure 6 shows the accuracies of Poria cocos powder obtained combining three combination preprocessing methods. The identification accuracy of adulterated samples is 100.0% with all combinations of three combination preprocessing methods. The cumulative variance contribution rates of first two scores with 2nd Der+MinMax+SNV and 2nd Der+MinMax+MSC are less than 71% and the identification accuracies of calibration dataset are unreliable. The best identification accuracy of calibration dataset is 93.8% with the 2nd Der+SNV+MinMax, 2nd Der+MSC+MinMax, SNV+2nd Der+MinMax, and MSC+2nd Der+MinMax methods. Although the accuracy of validation dataset with DT+MinMax+2nd Der is the highest (87.5%), the cumulative variance contribution rate of first two scores is less than 69% and the identification accuracy of adulterated samples is 0%. The best identification accuracy of validation dataset is 75% with 2nd Der+SNV+MinMax, 2nd Der+MSC+MinMax, SNV+2nd Der+MinMax, and MSC+2nd Der+MinMax methods. Therefore, the four combinations are the best preprocessing methods.

The selected four combinations are in order of baseline correction, scatter and trend correction, and scaling or scatter and trend correction, baseline correction, and scaling, which are basically the same as that reported in the literatures (Bian et al., 2020; Gerretzen et al., ,^2015^, ^2016^). The identification accuracies of the four combinations are the same. In addition, the results are poor without the strategy, even with different combination order. For example, the identification accuracies of calibration and validation dataset are 85.9% and 68.8% with MinMax+2nd Der+SNV, while the identification accuracies of calibration and validation dataset are 57.8% and 43.8% with MinMax+SNV+2nd Der. The results of these combinations are worse than those of the strategy. The results show that this strategy of “pick the best of the best” is not easily affected by the combination order. Besides, the number of combinations to be considered with the strategy is far less than that of random combination, which can reduce the computation and improves the efficiency.

**FIGURE 7:**
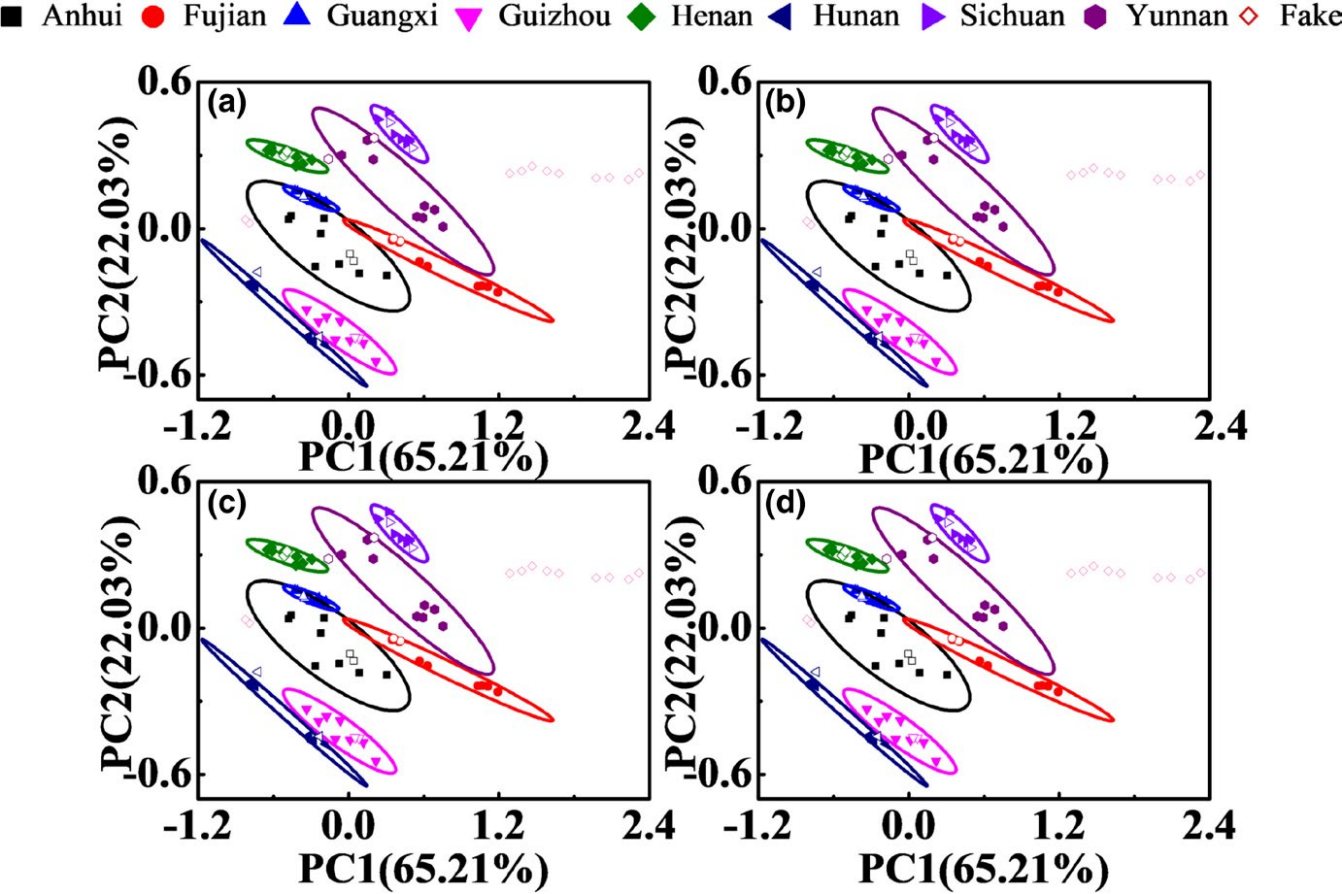
PCA results of Poria cocos powder and adulterated samples with the best preprocessing methods, (a)‐(d): 2nd Der+SNV+MinMax, 2nd Der+MSC+MinMax, SNV+2nd Der+MinMax, and MSC+2nd Der+MinMax

Figure 7 shows the PCA results of Poria cocos powder and adulterated samples with the best preprocessing methods. It can be seen that the results with all the best preprocessing methods are similar. The results by combining three preprocessing methods with “pick the best of the best” strategy are better than those without the strategy, which show that the strategy can significantly improve the model performance in a short time. The identification of Poria cocos powder and adulterated samples can be achieved by the strategy. However, the difference of Poria cocos from different producing areas is not significant, and is difficult to be identified. In addition, there is no need to provide prior knowledge of categories in PCA method, and the identification ability of PCA method is not very strong. 100% identification accuracy cannot be obtained with PCA methods and the supervised identification method using prior knowledge may be more effective.

## 4 CONCLUSION

In this paper, a novel strategy of “pick the best of the best” was proposed to obtain the optimized preprocessing method for the nondestructive identification of Poria cocos from different producing areas and adulterated samples with NIRS. Spectra of Poria cocos powder samples were obtained directly by the NIRS instrument. The optimized preprocessing combination can be obtained quickly with this strategy, and the identification accuracy can be greatly improved. 100% accuracy of adulterated samples, 93.8% accuracy of calibration dataset and 75% accuracy of validation dataset were obtained with the novel strategy. The developed technology can be regarded as a simple, rapid, and accurate nondestructive identification method for different‐origin and adulterated samples, and has a broad application prospect in the future.

## CONFLICTS OF INTEREST

The authors declared that they have no conflicts of interest to this work.

## Data Availability

The data that support the findings of this study are available from the corresponding author upon reasonable request.
